# Disparities in Healthcare Services and Spatial Assessments of Mobile Health Clinics in the Border Regions of Thailand

**DOI:** 10.3390/ijerph182010782

**Published:** 2021-10-14

**Authors:** Hiranya Sritart, Kuson Tuntiwong, Hiroyuki Miyazaki, Somchat Taertulakarn

**Affiliations:** 1 Faculty of Allied Health Sciences, Thammasat University, Pathumthani 12120, Thailand; somchat@tu.ac.th; 2 School of Dentistry, King Mongkut’s Institute of Technology Ladkrabang, Bangkok 10520, Thailand; kuson.tu@kmitl.ac.th; 3 Center for Spatial Information Science, Tokyo University, Chiba 277-8568, Japan; heromiya@gmail.com

**Keywords:** spatial accessibility, geographic information system, spatial distribution, healthcare disparity

## Abstract

Reducing the disparities in healthcare access is one of the important goals in healthcare services and is significant for national health. However, measuring the complexity of access in truly underserved areas is the critical step in designing and implementing healthcare policy to improve those services and to provide additional support. Even though there are methods and tools for modeling healthcare accessibility, the context of data is challenging to interpret at the local level for targeted program implementation due to its complexity. Therefore, the purpose of this study is to develop a concise and context-specific methodology for assessing disparities for a remote province in Thailand to assist in the development and expansion of the efficient use of additional mobile health clinics. We applied the geographic information system (GIS) methodology with the travel time-based approach to visualize and analyze the concealed information of spatial data in the finer analysis resolution of the study area, which was located in the border region of the country, Ubon Ratchathani, to identify the regional differences in healthcare allocation. Our results highlight the significantly inadequate level of accessibility to healthcare services in the regions. We found that over 253,000 of the population lived more than half an hour away from a hospital. Moreover, the relationships of the vulnerable residents and underserved regions across the province are underlined in the study and substantially discussed in terms of expansion of mobile health delivery to embrace the barrier of travel duration to reach healthcare facilities. Accordingly, this research study addresses regional disparities and provides valuable references for governmental authorities and health planners in healthcare strategy design and intervention to minimize the inequalities in healthcare services.

## 1. Introduction

Maximizing and achieving equity of access to healthcare systems is widely recognized as an important health policy goal of the overall population health in every country [1,2,3]. Providing access to quality and essential healthcare services is one of the key targets of the significant Sustainable Development Goals set out by the United Nations in 2015 as a comprehensive call to action on a global scale so that no one is left behind by 2030 [4]. Therefore, improvement in accessibility is considered a vital factor to achieve equity in the access to healthcare at both the local and regional levels toward achieving the SDGs [5]. However, identifying and measuring access to healthcare are quite challenging and complex; therefore, when developing a health policy, many nations still face a major challenge in delivering healthcare services in terms of enhancing accessibility [6,7,8,9,10].

Mobile health clinics are one of the crucial aspects that have recently been recognized to facilitate access to healthcare services that support and alleviate health disparities in difficult and underserved areas [11,12]. Mobile health clinics serve as a vehicle for inclusion and a platform for neglected regions by taking healthcare out of the hospital or doctor’s office and bringing it to the people [12]. It is a medical office on wheels, such as a van or a bus, or a recreational vehicle that has been modified and transformed into a self-contained space for medical treatment. Generally, the vehicle is designed and adjusted to comprise medical equipment, a waiting room and an exam room. The primary goal of mobile health clinics is to travel and visit communities where citizens do not have convenient access to clinical-quality healthcare. Several studies have revealed that this healthcare delivery model significantly expands healthcare accessibility and improves the health outcomes of the population by providing urgent care, preventive health screening and initiate and improve chronic disease management [11,13]. In the United States, there are an estimated 2000 mobile clinics that exist nationwide, receiving 6.5 million visits per year, based on the approximated 811 mobile clinics currently registered on the Mobile Health Map project [14,15]. These healthcare services serve communities that are located long distances away from any medical facilities or that lack transportation and have the poorest access to health services, including those of racial and ethnic minority backgrounds, the homeless, displaced populations, recent immigrants, migrant workers, people lacking insurance, or those from low socioeconomic status backgrounds [16]. Although mobile health clinics show the potential to help minimize healthcare barriers by increasing the healthcare services in remote areas, several concerns are continually discussed in terms of the limitations and challenges that must be addressed and overcome in order to establish a balanced model and efficiency of utilization [17]. One of the significant challenges around connecting the platform to the required demand of underserved regions is the lack of data [18]. The lack of accurate data for delivery service, particularly expanding the access to healthcare for hard-to-reach communities, needs to be significantly addressed to improve the delivery and sustainability of care [13,17,18,19,20].

Since access to healthcare is recognized as an essential facilitator of overall population health, the concept of access is complex and multi-faceted, involving various dimensions. Several aspects were discussed in previous studies. Five dimensions were proposed in 1981 by Penchansky and Thomas regarding access as more specific areas of fit between the population and healthcare, namely, availability (i.e., the sufficiency of healthcare services), accessibility (i.e., the relationship between location patients and providers), accommodation (i.e., the adequacy and suitability of healthcare services), affordability (i.e., healthcare utilization costs) and acceptability (i.e., compliance and satisfaction with healthcare services) [21]. While the first two dimensions of availability and accessibility are spatial, the last three groups are essentially aspatial, reflecting healthcare financial arrangements and cultural factors [22]. Thus, spatial access focuses on how healthcare accessibility is impacted by the distance variable, while aspatial access or non-spatial access highlights non-geographic barriers, such as age, sex, income and social class [23].

Regarding identifying the gaps in healthcare delivery and minimizing the disparity of health services, the geographic information system (GIS) has been applied to analyze healthcare needs, access and utilization in healthcare delivery for several decades [24,25,26]. The use of the GIS has received growing attention for connecting the diverse layers of the population and environmental data in order to analyze and characterize the many aspects of healthcare demand. In terms of recognizing vulnerable areas, geographical accessibility is widely recognized as a significant component in assessing a population’s overall access to healthcare and it is a fundamental goal for meeting the population’s health needs [27,28,29]. Previous studies have revealed that geographical barriers to healthcare access result in lower healthcare utilization, reduce the uptake of preventive services and decrease survival rates, which may contribute to poorer health outcomes, especially for those who live further away from healthcare facilities [30,31]. Furthermore, they are related to the inadequate utilization of specialized treatment, such as cancer screening and care, maternity and pediatric centers, frequently found in the large cities and not accessible for people living in rural areas and remote communities [32,33,34,35,36]. Commonly, applying the GIS for measuring geographic access to healthcare can be evaluated in terms of either area or by distance [25]. Area-based measures describe the ratio of population need to available services for areas such as countries, towns, or states. Several spatial analytic techniques have been proposed for describing and examining the relationship between different layers of population need combined with predefined geographical areas such as countries, zip codes, or municipality levels and for exploring how healthcare delivery can be improved [37,38]. Provider-to-population ratios are the most popular approach and have long been used to describe geographical disparities in access to healthcare across the United States due to the straightforward computation for bordered areas combined with the indicators of health service capacity, such as the number of hospitals or physicians [39,40]. Nevertheless, these supply ratios still contain some important limitations, because several variations in accessibility within bordering areas are ignored by these ratios. Based on these common ratios, this approach implies that all physicians are created as having equal access and that all communities have the same health needs, regardless of demographics and other conditions regarding access; however, in reality, they are impacted by several factors, such as barriers or conditions to travel, particularly for specific vulnerable populations [21,41,42]. Furthermore, when predefined units are large, most area-based measurements cannot reflect important factors for smaller units within the neighborhood, which may evaluate differences in access within the area [43,44].

On the contrary, the distance-based approach concentrates on the distance, travel time and cost between the population and healthcare providers. Most prior works have used a straight line or a Euclidean distance to identify the service area and measure the spatial barrier between healthcare providers and residents [45,46]. Murad used the GIS to present and apply the straight line distance function combined with a driving distance of five minutes to present a health accessibility model for identifying service and non-service areas in Jeddah City, Saudi Arabia [46]. Still, previous studies have revealed that only the Euclidean approach might not reflect the truth in practical physical constraints of movement and transportation routes, thereby underestimating the real travel distance [47,48]. In contrast, the network distance is the physical travel path or road to reach the destination [49]. Therefore, network distance has a major influence on geographical access regarding transportation mode, road distance, or traffic that impact the travel time required to reach healthcare providers [50]. Furthermore, numerous researchers have recognized that determining access is notably related to the timely use of service according to need [51,52,53,54].

Due to the imbalance in the spatial distribution of those healthcare demands and resources, the obstacle of travel to access healthcare considerably requires further analyses. The concept of traditional accessibility describes the term as an aggregate opportunity that denotes the number of potential possibilities or destinations that can be reached from a given point in a given amount of time [55,56]. Although several studies have applied a radius distance from the Euclidean concept to reveal the basic accessibility, it might still not reflect the real-world aspects of reaching the destinations. Therefore, in terms of investigating and analyzing the accessibility model for this study, real road network analysis was applied instead of straight-line distance to calculate accessibility. In recent times, aging societies have become a significant concern and a major demographic challenge confronting various countries [57]. Seniors are well recognized as a high-risk group that often require more healthcare resources and visits to the hospital more than younger adults [58,59]. Across these diverse socioeconomic groups, research has shown that, although healthcare services are geographically available, the times and demand of services are insufficient for some vulnerable populations [25]. Furthermore, the areas with the worst access to healthcare are often the places where poorer people live. In a previous study, Peters et al. also discovered that people in poor countries tend to have less access to health services than those in wealthier nations as a result of geographical, ethnic and socioeconomic differences [2]. Many barriers have an enormous impact on access to health services, especially in remote areas, particularly in terms of physical distance or travel time to health facilities or the availability of health services [2,60]. The transportation challenges due to the distance from healthcare facilities pose significant access obstacles and adversely affect their use [61]. Recently, Costa et al. applied the GIS methodology to measure the disparities of geographical access across Portugal and discussed that the areas lacking healthcare accessibility were in the border regions of the country due to the distance barrier and the depopulation process in these areas [22]. Several studies have also revealed and discussed that the evaluation of healthcare access, which is limited in these areas, is significantly required for precise assessment [62,63,64].

Based on the literature and on the principle of equity in healthcare access, people should have equal access to health services. In practice, however, this is quite challenging to achieve due to the limited medical resources, such as healthcare facilities or personnel in the healthcare profession, and geographical barriers. Mobile health clinics that offer care opportunities in underserved areas have the potential to minimize the gaps in access in terms of distance from health facilities, cost barriers and lack of transportation. However, no previous literature has taken this service into account to assess and enhance the accessibility of healthcare. To narrow the disparities in healthcare access and to improve the services of mobile health clinics, particularly for vulnerable populations and regions, a crucial initial step is, consequently, to identify the location of underserved populations to formulate suggestions for meaningful and effective government policy and local planning [37,65].

Therefore, the objectives of this study are as follows: First, to identify and assess the spatial distribution of healthcare in terms of the demands of the population and the services of the healthcare facilities in remote provinces, since, based on the previous literature, no study has yet investigated the border region and remote province of Ubon Ratchathani, Thailand. An additional purpose is to further analyze the spatial accessibility of healthcare based on the real road network and the travel duration for a realistic approach. Lastly, identifying the underserved area for which healthcare accessibility can be improved by mobile health clinics is the final goal, with the ultimate objective of providing recommendations to the local authorities or healthcare practitioners regarding reductions in the effect on the health disparities of the study area.

## 2. Materials and Methods

### 2.1. Methodology

Efficient planning and healthcare provision for public services are crucial for ensuring that the resources and capacity serve the purpose of reducing community costs and maximizing the benefits for people. Thus, understanding the location of the facilities and distribution between healthcare resources and population needs is urgently required for effective health service development and planning. The GIS technology is a significant tool that helps researchers understand critical information by visualizing it through mapping. However, analyzing and evaluating the accessibility of healthcare based on the disparities of the spatial distribution of population demands and healthcare resources is a significant issue that requires further investigation and discussion.

**Figure 1:**
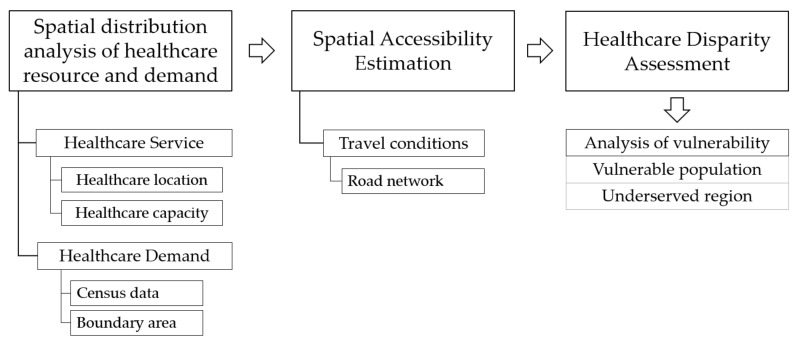
Methodology flowchart of the study.

Based on the previous literature, healthcare accessibility depends on three significant aspects, namely, healthcare service, potential demand and the capacity between the demand to reach the service [57,58,59,63]. Therefore, our assumption to expand healthcare delivery by mobile health clinics in the study area is affected by the disparity of the traditional public healthcare distribution, which alters the population demand and resources of healthcare. According to these issues, the condition to access healthcare, which reflects the cost and travel time, could be concealed in the summarized statistical data and suppress the underserved area in terms of access to mobile health clinics. To investigate these assumptions, we organized the method in the following steps (Figure 1): first, measurement and evaluation of the spatial distribution based on the demand and resources of healthcare in the study area; second, analysis of healthcare accessibility in terms of the travel conditions, particularly the road network; lastly, evaluation of the healthcare disparity and analysis of vulnerable population and regions. The details of the methodology are described in the following sections.

#### 2.1.1. Spatial Distribution of Healthcare Resources and Population Demand

Recognizing the imbalance in the amount, resources and distribution of both population and healthcare facilities is necessary for the public health planning and resource allocation. According to the previous literature, the visualization of spatial data is a significant stage in obtaining the existing conditions for further spatial data analyses [46]. Therefore, an area-based mapping method was applied to obtain the information and the spatial distribution was analyzed. The original administrative unit of the province polygon was used and divided into the smaller area resolutions; in this research study, we used a district area level. To explore the population data in the study area polygon, census data were joined to those smaller area polygons and merged to the district area with the same area code. To investigate the spatial distribution of healthcare resources, hospital list data were first geocoded to convert the addresses of the study into x- and y-coordinates, which were then validated visually for further analyses. These geocoded healthcare facilities were then joined with the health personnel data for each hospital.

#### 2.1.2. Spatial Accessibility Estimation

**Figure 2:**
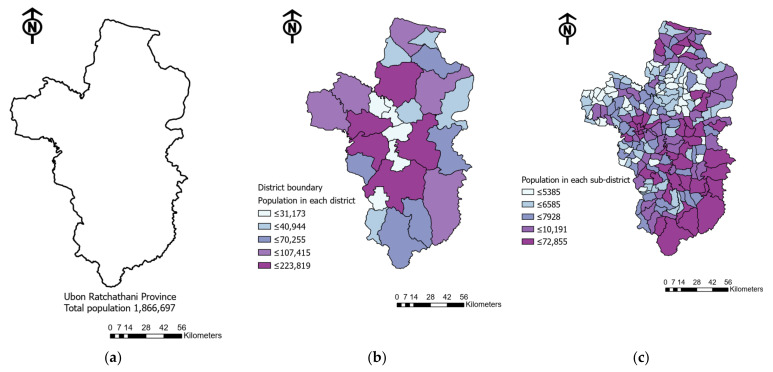
Area-based mapping of the research study: (**a**) original administrative unit scale of the province polygon; (**b**) polygons divided into smaller areas at the district level; (**c**) finer scale of sub-district-level polygons.

The original administrative units of the province scale might not reflect the dynamic distribution of the actual population within the boundary, nor the distribution of healthcare facilities for precise analysis, as per the intention of the study to identify the specific underserved area for local authorities to improve their strategic health plan. Based on the administrative boundary of the country, there are four levels in Thailand: country, province, district and sub-district levels. Therefore, the area-based mapping method in this study divided the province boundary into smaller-scale resolutions to acquire information and to estimate the spatial accessibility, as shown in Figure 2.

To represent the population point within the area on a finer scale, the district resolution was further divided at the sub-district level in order to contain the better resolution of the analyses. The centroid feature of each sub-district polygon was then generated and represented as the residents in this finer area level. Extending the travel route calculations and cost estimations, a road network analysis was generated and performed in this phase, using the GIS platform (ESRI, Redlands, CA, USA), to establish the path between each representative population point and location of healthcare facilities. Because the availability of healthcare services should be able to reach communities in life or death or emergency situations, we employed a closest facilities analysis, which is based on Dijkstra’s algorithm, by constructing two types of path-finding algorithms to find the shortest routes to the destinations [66]. To find the shortest exact path, the algorithm first calculates the distance between the starting point and each subsequent vertex until it reaches the destination point. Then, the second type is a hierarchical path solver for a possible faster and shorter route. By implementing the closest facilities analysis, as a destination point, the healthcare locations were used as the facility features in the GIS application, while the generated population centroid points were imported as the incident features for the starting position to calculate the route. After a network dataset was created to construct multimodal routes, travel directions, closest facilities, service areas and origin destination cost matrices, we employed healthcare locations as a destination point in implementing the closest facilities analysis in the GIS application. Meanwhile, the generated population centroid points were imported as the incident features for the starting position to calculate the route. The car travel mode was applied to perform the network analysis of this study.

Based on the accessibility being strongly constrained by the road network, the travel costs along the route from each population point to the nearest hospital, as well as the distance and duration of the route, were investigated to measure the accessibility. The road network distance between each point was computed, including the estimated travel time. Then, the population point within the threshold travel duration appropriate for the case study area was distinguished. As the primary output of this process, a table of each population point within the sub-district was created and the travel distance and time were evaluated. In this investigation, the car driving mode was considered the primary method for accessing the closest medical facilities, since cars are the most used vehicle by Thai citizens, even in rural areas [67,68]. Furthermore, based on the latest official regulations of vehicle speed on national or rural roads in Thailand, which states that the speed of small cars shall not exceed 120 km/h [69], the speed limitation for the network analysis in this study was set at 120 km/h.

#### 2.1.3. Healthcare Disparity Assessment

According to the previous literature, travel time is a significant and meaningful indicator in spatial accessibility to health facilities [70,71,72]; therefore, with regard to the evaluation of healthcare disparity, this paper calculated healthcare accessibility through a combination of access travel time by car and those spatial distributions of the population. In order to estimate the population in each area based on travel time accessibility, first, the travel duration was classified into three levels of accessibility: within 15 min, 15–30 min and 30–60 min. Then, the accessibility of the population to the closest healthcare facilities was calculated by combining the total amount of population that reach the facilities using the following Equation (1):

(1) $$R_{t}=\sum P_{s,t},$$

where $R_{t}$ is the estimated number of residents that reach the healthcare facilities within time t (in min) and P_s,t_ is the amount of the population in each sub-district area, s, that can access the nearest hospital within time t. This approach defines t as 0–15 min, 15–30 min and 30–60 min, which indicates that the travel time occurs within these parameters.

Regarding the evaluation of the vulnerable population based on the imbalance of spatial distribution, the measurement was calculated using the following Equation (2):

(2) $$R_{t}^{o}=\sum P_{s,t}^{o},$$

where $R_{t}^{o}$ is the estimated number of senior residents aged over 60 years old who can reach healthcare facilities within time t (in min) and $P_{s,t}^{o}$ is the amount of the senior population aged over 60 years old in each sub-district area, s, that can access the nearest hospital within time t.

In addition, to acquire and classify the underserved population, the ratio-based travel duration of residents to healthcare facilities was calculated as Equations (3) and (4):

(3) $$F_{i,t}=\sum {\;P}_{i,t}/P_{i},$$

(4) $$F_{i,t}^{o}=\sum P_{{\;}_{i,t}}^{o}/P_{i}^{o},$$

where $F_{i,t}\;$and $F_{i,t}^{o}\;$are the ratio of the estimated population and estimated seniors, respectively, within time t of each district i, whereas $P_{i,t}\;$is the calculated population of district i within time t and $P_{{\;}_{i,t}}^{o}$ is the computed number of seniors of district i within time t.

Finally, to identify and evaluate the underserved area in accessibility to healthcare, the accessed area of each district region was calculated using the following equation (5):

(5) $$S_{i,t}=\sum A_{i,t}/A_{i},$$

where $S_{i,t}\;$is the ratio of access area within time t of each district i and ${A\;}_{i,t}\;$is the estimated area within district i that accesses the nearest hospital within time t.

In order to address and recognize the particularly underserved area of healthcare accessibility, the relationships within the study area were investigated. According to the objective of the study to address the vulnerable senior population and accessibility in each sub-district area, the following four factors were considered and analyzed: travel duration, sub-district area, size of the population and size of the senior population within the sub-districts. A multivariate approach was applied to account for multiple variables partitioned into clusters.

### 2.2. Study Area

**Figure 3:**
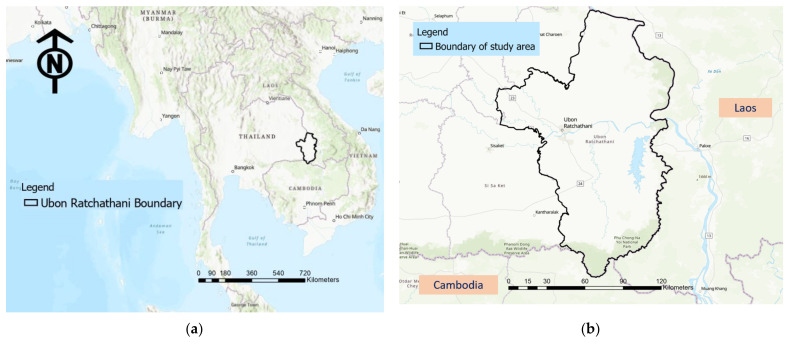
Location of study area: (**a**) Ubon Ratchathani in Thailand; (**b**) study area surrounded by the neighboring countries of Laos and Cambodia.

The study area of this research is an important province named Ubon Ratchathani, located in the northeastern region of Thailand. This town comprises one of the biggest provinces in Thailand and has the second-largest area in the northeast region. According to the official statistics registration systems in 2017, this study area has the third-largest population in Thailand of over 1.8 million residents, with a population density of 115.3 people/km^2^ [73]. However, geographically, the province is located in a remote area, being the easternmost province in the country, approximately 630 km from the capital city, Bangkok. Consequently, this province is almost completely surrounded by the neighboring countries—Laos from the northern to the eastern border and Cambodia along the southern borderline of Ubon Ratchathani, as shown in Figure 3.

Regarding the national report of older persons in Thailand and the significant concerns, Thailand has now become an aging society, which means that the number of elderly people is constantly increasing by over 10% of the total population. In short, the country is turning into a complete aged society, with more than 20% of the population being seniors [74]. Concerning these issues, Ubon Ratchathani is one of the major provinces facing the challenging situation of an increased elderly population. According to the official demographic data of the province, the ratio of senior residents continuously rises each year; particularly, the proportion raised to 13.1% in 2017 [73].

Due to the substantial increase in the elderly population, several reports were highly concerned about the improvement in healthcare delivery and planning. Screening and assessing the risk of geriatric diseases and analysis of changes in the health status of the elderly to prevent disability and promote self-help to live in society are required. However, challenges in the care and treatment of these residents still exist because of the obstacles of the costs of travel and distance between residents and hospitals [75,76].

Generally, most hospitals in Thailand are required to set up primary care units to take basic responsibility for the population in their surrounding catchment areas [77]. According to reports, healthcare facilities can be easily reached within 30 min in most parts of Thailand, whereas, in several parts of the country, the geographical accessibility of health services has a direct bearing on the utilization of these services [78,79]. However, only limited studies have investigated the geographical accessibility of healthcare services that impact their utilization [78].

Since the infrastructure of the road network in Thailand has been extensively developed, Thailand has 390,000 km of highways [80] and, according to the BBC, Thailand has 462,133 roads and many multi-lane highways [81]. Therefore, according to statistical reports, Thailand’s car ownership rate is relatively high compared to that of its neighboring countries. In 2019, an estimated 60.17% of Thai respondents reported owning a car [82]. The total number of registered vehicles in Thailand in 2020 was over 41 million vehicles [83]. 

The country began to emphasize health delivery infrastructure in the 1970s [84]. However, the first healthcare program of universal healthcare coverage to improve financial access was initiated in 2002. Numerous studies have debated and discussed the effectiveness of its design and implementation in reaching the population [85,86,87]. Furthermore, even with the universal healthcare access model deployed in the country, geographic disparities of healthcare facilities still exist and require further discussion and assessment, particularly in remote areas [88,89]. Therefore, Ubon Ratchathani is an appropriate location for conducting a study to identify underserved areas in geographic accessibility for potential mobile health clinic improvement that can later be extended to other regions of the country.

### 2.3. Collected Data

As regards efficient evaluation in accessibility, the aspect of the spatial distribution of both the population and health facilities must be considered. Therefore, the data applied in this study were obtained from several databases. The three main basic data required for the study were (1) the population data, (2) the boundaries of the study area and (3) the healthcare data and facilities of the area. Detailed information about the data, sources and application in this research study is presented in Table 1.

The population data of this study area were obtained from the official statistics registration systems, which collect information and provide census data by age range [90]. The population data in 2020 were collected in a .txt file for further processing. According to data availability, these data were acquired based on the administrative units of the province and also by small-area levels, which, for this study case, were at the district and sub-district resolutions.

The boundaries and the geographic data used in this study were obtained from mitrearth.org, which is an organization that supports and promotes the use of geological data [91]. The boundary of the study area was then collected on the province scale and the district polygon in the .SHP-file format was used as the basis for further processing.

The healthcare in Thailand is classified into three levels of different administrative scales and scopes of services, namely, primary, secondary and tertiary care. The primary care service provides healthcare services for populations on a sub-district level such as health promotion and disease prevention. However, at this level, there are no doctors working on a regular basis; therefore, the services only rely on the cooperation with doctors in the community hospitals. On the contrary, public or community hospitals are government-delivered healthcare services, which are under the Ministry of Public Health, who provide secondary and tertiary healthcare to the population at the district level. Therefore, in terms of analyzing the healthcare accessibility in this study, public hospitals were the focus of the study approach, because they are supposed to provide health services to those populations on their small-area level. As for the healthcare information required for this study case, the hospital list of Ubon Ratchathani was collected from the Open Government Data of Thailand site in a CSV file [92]. These data were officially provided by the Ministry of Public Health. The healthcare personnel lists of 2020 of the study case area were also obtained from the Ministry of Public Health. The data were offered by the Health Data Center, which is the central portal site that assists and encourages the public and researchers to utilize the government’s big data [93]. 

**Figure 4:**
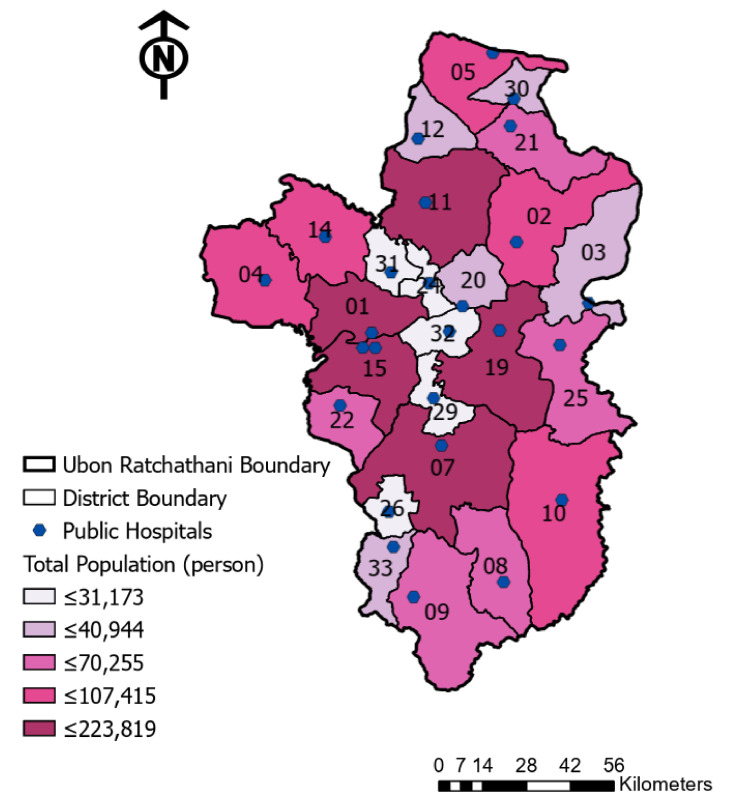
Spatial distribution of the public hospitals and population in the study area, Ubon Ratchathani.

In this research study, ArcGIS 10.7.1 (ESRI, Redlands, CA, USA) was utilized to process and map the geographical distribution and to analyze the spatial distribution of healthcare. The spatial distribution of the hospitals and the residential population of the study area is demonstrated in Figure 4. Although the distribution of the hospitals is almost even for each district, at least one hospital is located within each district region in Ubon Ratchathani; however, the distribution of the population in the districts is exceedingly diverse.

## 3. Results

The data of the study area are summarized in Table 2. For the total population in Ubon Ratchathani, only 26 facilities provide secondary and tertiary healthcare services, as a public hospital is located within this study area. According to the population data in 2020, the number of senior populations aged over 60 years old in the area has significantly risen and reached 14.94% of the total population. Because the overall area of this town is very large, covering an area of 15,484 km^2^, there is a significant requirement to analyze and illustrate the spatial distribution of the healthcare facilities and the demands of the population.

Exploring these statistical data with the GIS application, the disparity of the population is clearly illustrated in the visualized map. As illustrated in Figure 4, the distribution of the residents in the study area is extremely uneven between each district. Regarding the visualization of the diverse distribution of these population demands for healthcare, the classification of the residents was divided into five classes by a quantile method and illustrated in a different color, from white (small population) to dark purple (large population).

Comparing each administrative region at the district level, the largest population is distributed across three regions located in the middle of the west side of Ubon Ratchathani, indicated in dark purple (districts 1, 7 and 15). Meanwhile, the smallest population is located within 10 small districts that are displayed in bright white in the northern, middle and southern regions of the study area.

### 3.1. Spatial Distribution Analysis of Healthcare

**Figure 5:**
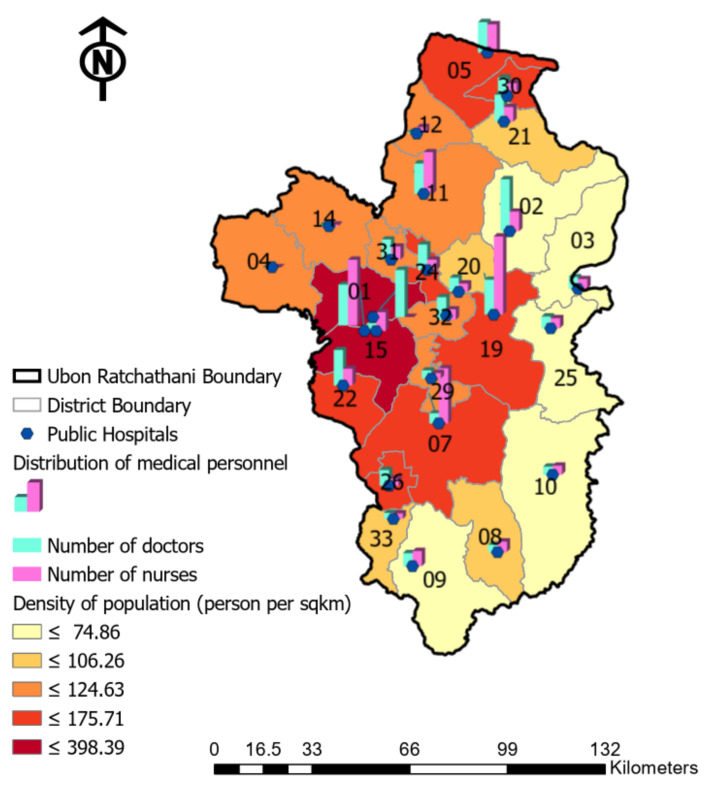
Spatial distribution of physicians and the population density within the study area.

Understanding the demand and resources of healthcare is significant for health planners and the decision-making processes to comprehend the barriers that exists in their communities. Still, the improvement in health outcomes that targets healthcare access cannot be achieved if vulnerable populations do not have access to skilled personnel or the necessary staff. Therefore, the spatial distribution based on the density of the population compared to healthcare resources, demonstrated in Figure 5, indicates that the inequality between demand and the availability of healthcare in the study area consists of an unequal distribution of not only facilities, but also the capacities of health personnel. Regarding the urbanization of Ubon Ratchathani, the concentration of residents in each territory is illustrated by the contrast in the availability of healthcare. 

As displayed in Figure 5, the greatest population density is contained in only two regions: districts 1 and 15, with over 398.39 people per square kilometer (dark red color). In contrast with the areas located around the eastern border, the density of the population within these remote districts clearly indicates a low resident concentration, illustrated in contrasting colors of light yellow. In addition, the spatial distribution of medical personnel, which indicates the key capacity resources between physicians and nurses in each district, is also demonstrated. Although the total capacity of medical staff in this study area encompasses 2372 doctors and 2206 nurses, once the analysis in the smaller administrative level is considered, the deficiency in healthcare capacity is revealed, as presented in Figure 5.

The average number of physicians for each district in Ubon Ratchathani is 90, with a median value of 66, whereas the distribution of doctors fluctuates from 0 to 243. As per this distribution demonstrated in Figure 5, these physician capacities are allocated unevenly for each district, with the highest physician capacities in the middle and northern areas (high symbol of the green bars). Comparing the nurse capacities, the minimum and the maximum number of nurses in the study area varies between 0 and 366. The spatial disparity based on the number of nurses is visualized in the different scales of the pink bar, indicating that the high capacities of nurses are located mostly in five areas only, which are districts 5, 7, 11, 15 and 19 (tall pink bar), whereas the other regions have low or nearly invisible levels.

### 3.2. Spatial Accessibility Analysis

According to the literature, proficient healthcare design and delivery require an essential understanding of the geographical conditions, the spatial distribution of the healthcare capacity and the demand of the population. Determining the underserved area and population via further accessibility analyses is essential.

**Figure 6:**
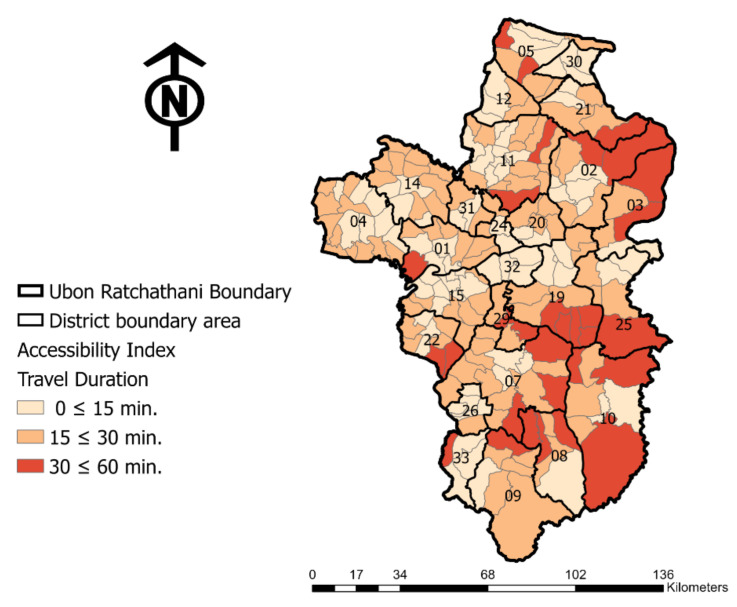
The accessibility index for each sub-district area based on accessed travel time duration.

For the accessibility analyses, the results using the smaller-area level in this study area were explored to illustrate the accessibility index based on the travel duration. Table 3 shows the estimated number of areas and population with the distinct healthcare accessibility of Ubon Ratchathani. From the total 219 sub-district area levels of the province, the results based on the road network analysis show that, across almost 24% of the region, covering 30 sub-districts, residents must travel over 30 min to access a hospital. Meanwhile, in most areas of the 189 sub-districts, people are able to travel to a hospital within 30 min (Figure 6), although the analysis revealed that 45.2% and 41.2% of the total population in the study area are able to access a hospital within 15 and 30 min, respectively. However, 13.6% of the total population (an estimated 253,802 residents) have to travel more than 30 min to reach a hospital. Relatively to these numbers, 13.2% is the population aged over 60, which reflects 33,534 seniors.

### 3.3. Healthcare Disparity Assessment

To present the differences in the access to healthcare facilities and the availability of services, Figure 6 demonstrates the accessibility index in each sub-district area in three levels based on a travel duration of within 15, 15–30 and 30–60 min to reach the hospitals in different light orange to dark red colors. The long travel duration indicated in dark red areas, reflecting the areas with less access to hospitals, is mostly located in the eastern region of the province. As revealed in the figure, most of the long travel durations to access a hospital are located in the eastern region of Ubon Ratchathani, which is connected to the border of the neighboring country of Laos. Meanwhile, in the middle of the province and in the northern area, most of the people in these regions are able to access a hospital within a short travel duration.

Table 4 below summarizes and presents the outcome for each district of the study area based on the disparities in spatial distribution and the accessibility index. The results of the estimated area using the road network analysis show that the regions with a low level of access are located mostly in the districts located along the eastern border. In particular, the residents living in districts 10, 3, 25, 2 and 8 are challenged in terms of access to healthcare facilities due to the long travel duration, which covers 60.9%, 53.9%, 41.2%, 40.0% and 35.5% of the district area, respectively.

**Figure 7:**
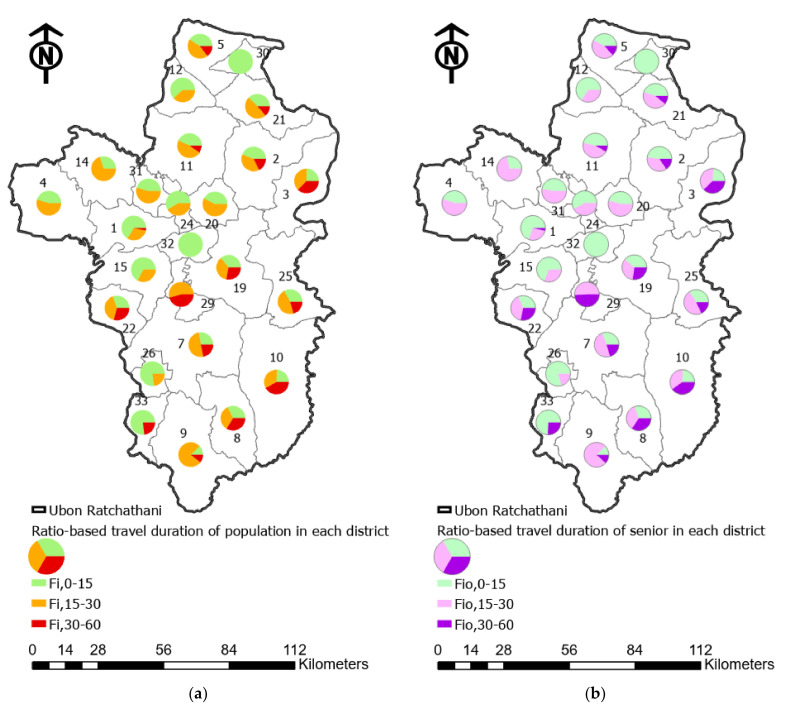
Accessibility levels of each district in Ubon Ratchathani by (**a**) the ratio of the estimated population and (**b**) the ratio of the estimated senior population.

To address the increasing elderly issue in the study area, Table 5 reveals the ratio of the estimated populations, particularly the proportion of the seniors within each district that must travel to reach public hospitals based on the three different levels of travel duration, as demonstrated in Figure 7 below. The results show that over 30% of the total residents in several regions has to travel more than 30 min to reach healthcare facilities (districts 29, 10, 3 and 8, with percentages of 45.5%, 41.7%, 37.3% and 33.6%, respectively).

Furthermore, in these areas, the percentile of senior residents who have to travel more than 30 min also reflects the high degree of low accessibility outcomes, as shown in Figure 7 below. On the contrary, Table 5 outlines that populations in the 10 regions with high access levels to a hospital with less than 30 min travel time, which are district numbers 4, 12, 14, 15, 20, 24, 26, 30, 31 and 32—particularly districts 30 and 32, where the entire resident population is able to drive to a hospital and reach it within 15 min.

**Figure 8:**
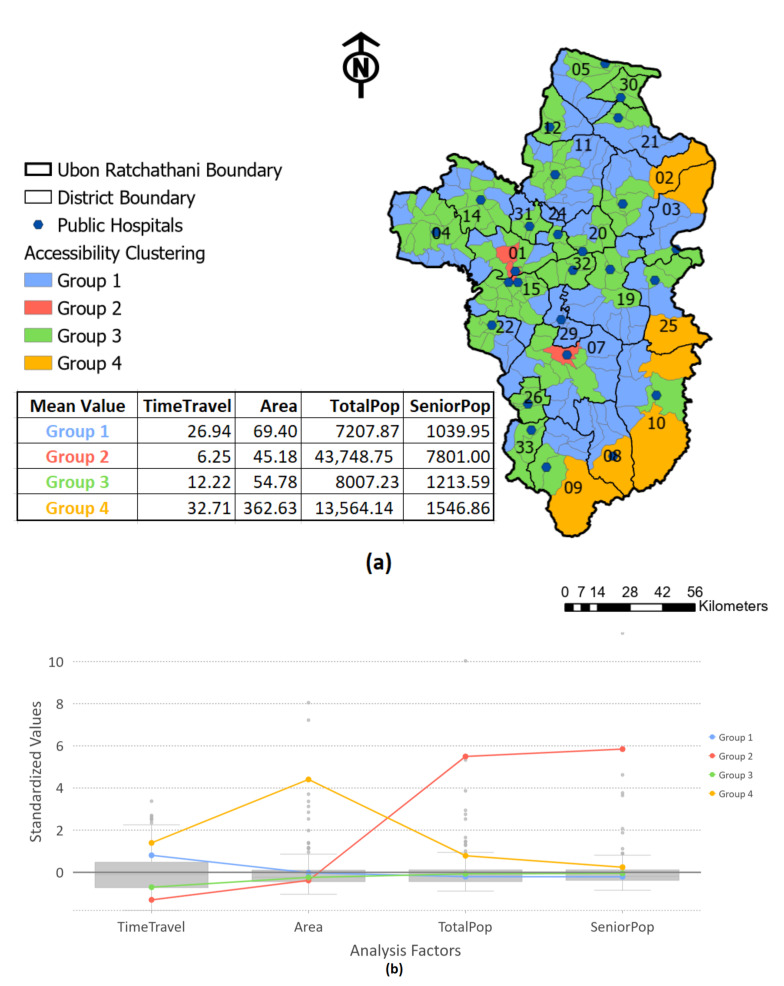
Spatial clustering analysis based on travel duration, covered area of each sub-district, total population within the area and demand of the senior population: (**a**) four groups of classification in Ubon Ratchathani; (**b**) box-plot of the clustering comparing the distributions of different data variables.

To further explore the relationships among the analysis factors within the study area in order to address and recognize the particularly underserved areas of healthcare accessibility, a clustering analysis based on the travel duration, the smaller administrative level areas and the number of demands from the population, particularly senior residents, was included into the analyses. Four groups of clustering were calculated using a k-means algorithm, as demonstrated in Figure 8. Additionally, the results of the properties of each group, as well as the characteristics of each variable utilized in the analysis, are displayed in box plots in Figure 8b. The *R*^2^ value was also computed for each variable and is reported in Table 6. The *R*^2^ value reflects how much variation of the analysis factor in the data was retained after clustering; the higher the *R*^2^ value for a specific variable, the better that variable is at distinguishing among the features.

According to the results from the clustering analysis, four groups were classified with different colors, namely, blue, red, green and orange, as shown in Figure 8a. Most of the sub-districts were classified into groups 1 (blue) and 3 (green), allocated around the province. Several areas in the middle of the study region are within districts 1, 15 and 17, categorized into group 2, whereas seven regions along the eastern border were grouped in orange. The results of the clustering explain that the residents within these specific, red-colored regions on the GIS map, group 2, are able to access healthcare services in a lower travel time; nonetheless, the concentration of population demand within these areas is significantly higher than in other regions. Compared to the results of group 4, the outcomes reveal that, according to the variable factors, these orange areas are the most vulnerable regions in terms of a low level of healthcare accessibility due to the longest travel duration and the largest covered sub-districts, including the highest population demand and, especially, the largest demand of the senior residents.

As illustrated by the box plot, the chart reveals both the clusters and the analysis factors within the chart. Notice that group 4 reflects the areas with the highest travel time and also the highest value for the covered area—with the number of population and seniors being above average, compared to the other groups. Meanwhile, group 2 reveals the areas with the greatest population, as well as the highest number of seniors, with the shortest travel duration to health facilities and also the smallest covered area. Group 3 indicates the area with the almost lowest travel time and covered area, which are not as low as group 2, with a lower-than-average number of the total and elderly populations. In contrast, group 1 shows the sub-district area with the almost highest travel time, less than the average of the covered area and the number of population and seniors, compared to other groups. The average values of each analysis factor are demonstrated in Table 6, including the minimum, maximum and standard values.

## 4. Discussion

### 4.1. Overall Assessment of Spatial Accessibility of Healthcare Facilities

Geographical and potential access remains a crucial element of healthcare utilization in times of need and health service planners and policymakers need to design and implement strategies to minimize these access difficulties regarding services. To support these local or nationwide authorities, it is essential to understand the environmental access conditions of the residents, particularly in remote regions, as well as to recognize the constraint of the drivers of vulnerable populations. Given the importance of an evidence-based plan, the findings of this study of remote regions, specifically border areas, contribute significantly to the literature.

The results presented in this article highlight the issue of spatial inequity of healthcare resources and the imbalance in demand of the population of the study case area, Ubon Ratchathani. We employed the GIS to measure the geographical access that integrates the real road network aspects in the research approach. Compared to the traditional measurement of the provider-to-population method, the results of this research offer more understanding and a method to identify underserved areas and vulnerable populations to expand the mobile healthcare services for those communities, as regards a reduction in the effect on the health disparities of the study area.

As present, GIS-based mapping is a significant tool to analyze and assess the spatial distribution of limited public hospitals and medical personnel, along with the disparity of the population within each district area. Relatively to the elevated number of resident demands, as well as the high density of the population, out of the overall results, districts 1, 15 and 7 presented a high classification rank compared to the other districts, particularly districts in the remote border regions. In addition, focusing on the effect of travel time to the nearest hospitals, this research study also underlines the challenging barrier that is to be overcome by mobile health clinics to improve healthcare access.

### 4.2. Disparity Assessment of Healthcare Service Distribution

First, based on this approach, our results reveal that 13.6% of the overall population in the study area need to travel more than 30 min to reach the nearest public hospital, as indicated in 30 sub-districts that cover 23.9% of the area of the entire province. Furthermore, these people live mostly in rural and border districts. The previous literature indicates that residents in the border area might lack accessibility in several dimensions, economic activities and particular services because they are located in poorly inhabited areas away from densely populated inner-provincial areas [37,94]. Our findings also confirm this statement and expand additional accessibility in the healthcare access aspect, particularly at the smaller-area level rather than the district level. Furthermore, based on our findings, it is recommended that other options of healthcare service, particularly mobile health clinics, should be coordinated and provided for communities to attenuate the problem of high travel costs and duration in these neighborhoods.

Moreover, the condition of travel to the nearest hospital was considered in this study based on travel duration. According to previous studies, the timely use of services in response to need is significantly associated with access [57,58]. Because hospital closures affect healthcare access and also health outcomes, studies have revealed that an increased distance to the closest hospital increases the number of deaths from heart attacks and unintentional injuries [95,96]. Therefore, our approach divided the travel time range within the golden hour rules of 60 min, which is the period that is frequently used to describe the critical need of patient care [70]. Prior studies have applied 30 min as a standard travel time to a hospital, depending on the incident, i.e., patients who are critically injured and are treated within time frame maintain the best chance of survival [71,72,97]. Regarding this, it comes down to the fact, for the study approach, that populations located over 30 min of a hospital should be used to evaluate healthcare accessibility. Our outcomes of healthcare access in Ubon Ratchathani revealed that, due to the long travel duration of over 30 min, residents in certain districts encounter a significant challenge in terms of driving to the hospital, particularly districts 10, 3, 25, 2 and 8, which are located along the Laos border. In remote areas, public transportation has been discussed in the previous literature, specifically in developing countries. Typically, the public transit system is in poor condition, with low accessibility and inadequate network coverage [68,98]. Mostly, several provinces in Thailand still face these challenges. The only public transport in Ubon Ratchathani is by bus, which has a limited on-time schedule and travel area. Although, considering the prior literature, what is accessible by private car may be inaccessible by public transportation. Our findings still imply the underserved area based on common transport by car and identify a vulnerable area that could be focused upon more by medical practitioners to reduce the timely demand in reactive healthcare of visiting the hospital and to enhance the proactive healthcare within these areas [99,100].

Frequently, the problem with the healthcare demand is profoundly linked to the population concentration due to the urbanization process in metropolitan centers and citizen aging. These issues have been seen in several countries [101,102]. Concerning accessibility at the national level, the certain administrative district stage must be considered, where the management of residents’ health is delegated to this specific region. Based on our outcome in the district and sub-district levels of administration, we also observed that, within these districts of a low level of healthcare access, the ratio of the senior population is considerably high, compared to other districts. Although the depopulation of total residents reflects the results in border districts, the number of senior residents in the study highlight the expected consequences concerning aging in rural depopulated areas. Therefore, on the basis of our statistical results and, evidently, the mapping of our findings, the underserved area or at-risk vulnerable population of the study area offers useful material for supporting city authorities and medical practitioners in planning to minimize the access gaps. Based on the research outcomes, it is recommended that mobile health clinics should be arranged to expand access to these vulnerable communities and increase population health management. Particularly within those sub-districts that contain a high number of seniors and facing the difficulties of long travel distance and time to healthcare facilities, providing mobile health clinics with easier access within walking distance or a short distance for vulnerable groups could be useful services to improve health disparities in remote areas in Thailand.

### 4.3. Limitations of This Study

There are also several limitations to the present study. First, based on the classification of healthcare access in the previous literature, this study emphasized potential access only concentrating on the age of the population due to the aging challenge of the study area. In future studies, other factors of healthcare utilization behaviors, including additional elements of socioeconomic status such as income and educational level, could be further investigated to reveal actual access measurements. Second, from the methodological perspective, the transportation mode applied to the approach is concentrated through the road network by car. Public transportation was not considered in this study area due to the lack of convenience, availability and inefficient or inadequate information provided for the rural area. Another limitation is that only the public hospitals within the study area were examined with regard to identifying healthcare access in the research study. Third, according to the intentions of this study to identify the specific underserved area for the local authorities to improve their strategic health plan in Ubon Ratchathani, this approach to identify and analyze the area in each sub-district, applying the centroid of the area to reach the nearest healthcare service, was considered. However, due to the large size of the sub-district boundaries and the population distribution within each district being numerous and varied, for future studies, the population-weighted time and smaller scale of the sub-district level can be applied to address the issue of the modifiable areal unit problem (MAUP) to enhance further analyses. A further perspective of healthcare evaluation could be to apply more than one dataset combine with other physical or mental healthcare facilities as a means to improve population health.

## 5. Conclusions

In this paper, we applied the GIS methodology for the accessibility analyses of provincial health service planning. The significance of using the GIS application was demonstrated as a powerful and effective communication tool to exploit and consolidate significant patterns and for the spatial visualization of accessibility when quantitative outputs are displayed. Due to the limited and inadequate healthcare services, along with the uneven demand of the population, this research study addresses these critical issues that impact accessibility and evaluates healthcare access in the remote and border regions of Thailand. Applying the GIS time-based approach with the real road network combined with smaller-area units to identify the low health accessibility regions in the study area, Ubon Ratchathani, according to our findings, certain parts of the town were notably considered as poor hospital accessibility zones, particularly several sub-districts in the eastern area of the province. Here, the relationship between vulnerable residents and underserved regions is presented and the perspective of potentially expanding the healthcare delivery of mobile health clinics for communities is emphasized. Additionally, our results highlight the current status of healthcare access in the study area; the GIS-based approach can be further extended to other regions and other perspectives of healthcare accessibility investigation.

In conclusion, we believe this research study offers valuable information and contributes to the local and national authorities for designing and implementing future healthcare efforts to minimize the barriers of access disparities.

## Figures and Tables

**Table 1:** Collected data for the study.

Data Information	Geographical Level	Year	Source	Data Type	Application
Population data	Province	2020	The official statistics of Thailand	Text file	Spatial distribution analysis
	District				
	Sub-district				
GIS-boundary data	Province boundary	2020	mitrearth.org	Shape file	Boundary of study area
	District boundary				
	Sub-district boundary				
Healthcare data	Province	2020	The Open Government Data of Thailand	CSV file	Spatial distribution analysis
Health personnel data	Province	2020	Health Data Center	CSV file	Spatial distribution analysis

**Table 2:** Summary of statistics data of study area.

Information of Study Area		Amount
Ubon Ratchathani information	Area (km^2^)	15,487
	Population density (people/km^2^)	120.53
	Number of district areas	25
	Number of sub-district areas	219
Population information	Total population	1,866,697
	Male	930,904
	Female	934,297
	Senior population	278,901
	Male	156,208
	Female	122,693
The lowest administrative level: sub-district	Min.–max. area (km^2^)	1.91–604.09
	Average area (km^2^)	70.72
	Min.–max. population	2816–72,855
	Average population	8524
Healthcare Information	Number of public hospitals	26
	Number of physicians	2327
	Number of nurses	2206

**Table 3:** Accessibility based on the estimated area and population to reach the nearest healthcare provide within a certain time period.

Access Duration (min) (t)	Number of Sub- district	Area (km^2^)	% Area	Estimated Population $\left(R_{t}\right)$	% Population	Estimated Senior $\left(R_{t}^{o}\right)$	% Senior Population
0 ≤ 15	85	4827	31.2%	844,393	45.2%	132,967	15.75%
15 ≤ 30	104	6964	45.0%	768,502	41.2%	112,400	14.63%
30 ≤ 60	30	3696	23.9%	253,802	13.6%	33,534	13.21%

**Table 4:** Summary of accessibility of areas for each district based on the travel duration to healthcare facilities.

District no.	Total District Area (km^2^)	Number of Sub- Districts	The District Population-Weighted Time to Reach Healthcare Facilities	Area Ratio of Each District Area (S_i,t_)		
			(min.)	$S_{i,0–15}$	$S_{i,15–30}$	$S_{i,30–60}$
1	561.8	12	18.74	34.1%	51.6%	14.3%
2	947.9	11	28.54	26.7%	33.3%	40.0%
3	718.4	5	33.22	16.3%	29.8%	53.9%
4	861.9	18	18.82	46.8%	53.2%	0.0%
5	603.0	9	23.11	40.4%	43.4%	16.2%
7	1260.7	16	26.85	12.9%	57.7%	29.3%
8	650.2	6	29.00	45.2%	19.3%	35.5%
9	1038.0	7	26.46	11.3%	79.8%	8.9%
10	1485.1	8	29.25	16.3%	22.8%	60.9%
11	1020.4	23	23.09	30.9%	53.1%	16.1%
12	346.9	5	18.98	61.5%	38.5%	0.0%
14	727.5	14	19.33	29.0%	71.0%	0.0%
15	613.5	16	16.12	47.2%	52.8%	0.0%
19	950.5	14	27.11	22.7%	46.1%	31.1%
20	311.6	6	16.86	27.2%	72.8%	0.0%
21	554.0	6	26.21	17.6%	58.2%	24.2%
22	388.4	9	25.15	24.8%	46.9%	28.3%
24	184.8	4	20.75	55.2%	44.8%	0.0%
25	766.7	6	25.05	23.1%	35.6%	41.2%
26	215.6	5	12.25	68.3%	31.7%	0.0%
29	248.1	3	29.15	0.0%	66.2%	33.8%
30	218.7	4	10.03	100.0%	0.0%	0.0%
31	230.5	4	15.98	43.7%	56.3%	0.0%
32	264.8	4	13.05	100.0%	0.0%	0.0%
33	318.2	4	21.43	84.4%	0.0%	15.6%

**Table 5:** Accessibility of each district to access healthcare facilities within time period t, distinguished by the ratios of estimated population and senior population.

District no.	Ratio of Estimated Population in Each District $\left(F_{i,t}\right)$			Ratio of Estimated Senior in Each District $\left(F_{i,t}^{o}\right)$		
	$F_{i,0–15}$	$F_{i,15–30}$	$F_{i,30–60}$	$F_{i,0–15}^{o}$	$F_{i,15–30}^{o}$	$F_{i,30–60}^{o}$
1	66.6%	30.0%	3.4%	69.3%	27.0%	3.7%
2	44.0%	38.4%	17.6%	47.7%	36.7%	15.6%
3	24.8%	37.8%	37.3%	24.0%	38.2%	37.7%
4	45.5%	54.5%	0.0%	44.3%	55.7%	0.0%
5	41.0%	44.2%	14.8%	42.3%	44.5%	13.2%
7	27.8%	50.6%	21.6%	30.8%	48.8%	20.4%
8	32.5%	33.9%	33.6%	30.5%	35.2%	34.3%
9	12.0%	77.4%	10.6%	11.9%	76.6%	11.6%
10	24.2%	34.0%	41.7%	24.5%	35.4%	40.1%
11	43.1%	47.2%	9.7%	46.6%	45.1%	8.3%
12	61.9%	38.1%	0.0%	65.5%	34.5%	0.0%
14	30.0%	70.0%	0.0%	27.8%	72.2%	0.0%
15	67.4%	32.6%	0.0%	68.5%	31.5%	0.0%
19	36.2%	35.8%	28.1%	38.9%	34.0%	27.1%
20	42.8%	57.2%	0.0%	45.6%	54.4%	0.0%
21	37.6%	48.8%	13.5%	44.9%	44.0%	11.1%
22	30.8%	39.8%	29.3%	31.2%	40.6%	28.2%
24	58.6%	41.4%	0.0%	57.9%	42.1%	0.0%
25	33.0%	46.4%	20.6%	34.1%	47.8%	18.2%
26	77.4%	22.6%	0.0%	79.8%	20.2%	0.0%
29	0.0%	54.5%	45.5%	0.0%	50.8%	49.2%
30	100.0%	0.0%	0.0%	100.0%	0.0%	0.0%
31	45.9%	54.1%	0.0%	47.6%	52.4%	0.0%
32	100.0%	0.0%	0.0%	100.0%	0.0%	0.0%
33	77.0%	0.0%	23.0%	74.3%	0.0%	25.7%

**Table 6:** The output of the clustering analysis based on the four different variables of travel duration, area of each sub-district, population within the area and number of senior residents.

Analysis Factor	Mean	Standard Deviation	Min.	Max.	*R* ^2^
Travel time	19.03	9.78	1.35	52.10	0.6338
Area	70.72	66.11	1.91	604.09	0.6563
Total population	8523.73	6398.12	2816	72,855	0.5943
Senior population	1273.52	1113.94	324	13,950	0.6492

## Data Availability

The data presented in this study are available on request from the corresponding author.
